# Grocery store workers’ knowledge, attitudes, and barriers influencing uptake of COVID-19 vaccine in the United States: a qualitative study

**DOI:** 10.1186/s12889-026-26684-y

**Published:** 2026-04-02

**Authors:** Harpriya Kaur, Nikie Sarris Esquivel, Julianne Payne, Becky Durocher, Karen Strazza, Jacqueline Sivén, Michael A. Flynn, Catherine Viator, Cammie Chaumont Menéndez

**Affiliations:** 1 National Institute for Occupational Safety and Health, Centers for Disease Control and Prevention, Cincinnati, OH USA; 2 Research Triangle Institute (RTI), 3040 E Cornwallis Rd, Research Triangle Park, Durham, NC 27709 USA; 3 Present Address: Office of Community Health and Hazard Assessment (OCHHA), Agency for Toxic Substances and Disease Registry (ATSDR), Centers for Disease Control and Prevention, Atlanta, GA USA

**Keywords:** Grocery store workers_1_, COVID-19_2_, Vaccines_3_, Barriers_4_, Occupational safety_5_, Essential workers_6_

## Abstract

**Objective:**

The objective of the study was to gain insight into the knowledge, behavior, attitudes and beliefs related to COVID-19 vaccines, and communication preferences of U.S. grocery store workers.

**Methods:**

In-depth interviews were conducted from May 2021 through June 2022 with 75 grocery store workers across the United States (US) who identified themselves as Asian, Black, Hispanic, or White persons. Researchers used maximum variation sampling to recruit a diverse sample by race and ethnicity, age, sex, union status, and geographic location. Rapid Turn-Around (RTA) techniques were utilized to conduct qualitative data analysis.

**Results:**

Of the 75 total participants, 39 were female and 36 were male. Participants identified as Asian (*n* = 16), Black or African American (*n* = 19), Hispanic or Latino (*n* = 16) and White (*n* = 22) persons and ranged in age: 18–34 years (*n* = 28), 35–49 years (*n* = 20) and ≥ 50 years (*n* = 27). Most (79%) participants reported that they were vaccinated against COVID-19 and considered various factors when deciding to get vaccinated such as protecting oneself and loved ones, vaccine safety and potential side effects, others’ experiences with vaccination, the vaccine development process, beliefs regarding their immune systems, vaccine effectiveness, and similarities between the COVID-19 and influenza vaccines. Most unvaccinated women expressed concerns about side effects, compared to about half of the unvaccinated men. Specifically, most unvaccinated female workers had concerns related to fertility issues. Participants’ attitudes regarding employer vaccine incentives and mandates and sources used to learn about the vaccine varied by vaccination status. Although participants’ employers had provided them information regarding the COVID-19 vaccine, these communications were not in participants’ preferred formats.

**Conclusion:**

Unvaccinated participants’ attitudes and beliefs suggest there is a critical opportunity to address misinformation related to COVID-19 vaccines and potential side effects among grocery store workers. This study provides evidence of a gap for culturally and linguistically appropriate health communication efforts that are aligned with grocery workers’ health communication preferences.

**Supplementary Information:**

The online version contains supplementary material available at 10.1186/s12889-026-26684-y.

## Introduction

 On March 13, 2020, when the United States (US) declared COVID-19 a national emergency, most states began to issue stay-at-home orders [1]. However, essential workers, including approximately 2.7 million grocery store workers [2], were exempted from quarantine measures and continued to work, placing them at increased risk for workplace transmission of SARS-CoV-2 infection [3]. Grocery store workers were designated as an “essential critical infrastructure workforce” by the Cybersecurity and Infrastructure Security Agency at the beginning of the pandemic [4] and later listed in Phase 1b under essential non-healthcare workers for vaccination as prioritized by the Advisory Committee on Immunization Practices [5]. Of all the frontline workers who were prioritized for COVID-19 vaccine, grocery store workers were among those with the lowest wages, the least protection from SARS-CoV-2 infection, minimum training, and most limited access to employer-sponsored benefits like health insurance, paid sick leave, and hazard pay [6]. Grocery store workers are at heightened risk of COVID-19 due to social and health inequities [7, 8]. Moreover, the interactive nature of their job puts these workers at greater risk of contracting the infection from customers and co-workers [6, 9, 10]. A study conducted in Massachusetts in May 2020, early during the pandemic, found grocery store workers in customer-facing roles were five times more likely to test positive for SARS-CoV-2 than their co-workers in other positions [11].

According to a 2020 report that utilized 2014–2018 American Community Survey data, nearly 38% of grocery store workers were people from racial and ethnic minority groups, and about 27% were aged 50 and above [12]. As the pandemic evolved, reports indicated how COVID-19 disproportionately impacted older people and people from racial and ethnic minority groups [13, 14]. People from racial and ethnic minority groups such as Asian, Black or African American (hereafter referred to as Black), and Hispanic or Latino were at much greater risk of COVID-19-related hospitalization and death compared to people from white racial groups [15]. Although frontline essential workers, including grocery store workers, were prioritized second only to healthcare workers during the phased distribution of the COVID-19 vaccine, vaccine hesitancy among workers remains [16]. Many of these workers have lower educational attainment, may come from communities that have historically been underserved by public health institutions, or may come from communities that have experienced medical exploitation, unconsented experimentation, and social and economic marginalization, all of which may contribute to lower acceptance of vaccination [17, 18]. Since racial and ethnic minority groups in the U.S. had higher rates of SARS-CoV-2 infection, hospitalization, and deaths, it is crucial for workers living in communities with an increased burden to be vaccinated [19]. Lack of effective vaccination strategies equitably employed to reach populations at higher risk for SARS-CoV-2 exposure may have further exacerbated the existing health inequalities and continued to marginalize racial and ethnic minority groups [20].

The COVID-19 vaccine is the primary method to prevent severe COVID-19 since it has become available [21]. However, for an immunization program to succeed, it is paramount to have a high rate of acceptance and coverage [22]. Along with that, the population must have access to reliable information and services to make informed decisions. Unfortunately, the COVID-19 pandemic was accompanied by widespread misinformation about the virus which contributed to concerns regarding vaccine effectiveness and safety, as well as fear of potential side effects; this impeded the effectiveness of the public health response by creating distrust among the general population [23, 24]. Attempts to mitigate the disease were further complicated by the limited capacity of public health agencies to address the needs of a racially, ethnically and culturally diverse workforce of grocery and retail workers deemed critical especially during the public health emergency [18].

Identifying strategies to enhance vaccine uptake are still needed as new variants of the SARS-CoV-2 virus continue to emerge and updated vaccinations remain necessary to protect workers from serious effects of COVID-19 [25, 26]. Most of the research related to workers’ vaccine uptake to date is limited to healthcare workers; very few studies have focused on grocery store workers in the U.S [16, 27]. The purpose of this qualitative study was to understand grocery workers’ knowledge about COVID-19, their attitudes toward the vaccine, and the barriers influencing the uptake of the COVID-19 vaccine. Findings are critical for development of effective health communication approaches that are tailored to knowledge, attitudes, and cultural beliefs of workers and that address vaccine hesitancy among grocery store workers. Guided by World health Organization’s 3 C’s framework for vaccine hesitancy that focuses on confidence in vaccine safety and effectiveness, complacency related to perceived risk of COVID-19 and convenience in accessing vaccination, this study examines how individual, structural and informational factors shape workers’ attitude and behaviors towards vaccines and identifies the resources workers used to access reliable information related to COVID-19, and their preferred modes of communication.

## Materials and methods

### Study design and population

For this study, individual in-depth virtual interviews were conducted from May 2021 through June 2022 with 75 grocery store workers in the U.S. to learn about their experiences as public-facing essential workers during the pandemic. Of relevance to the current study, participants reported on COVID-19 vaccination status and vaccine beliefs, sources of information about COVID-19 vaccines, whether the employers provided an incentive to be vaccinated, whether the employer required employees to be vaccinated, and their feelings about a vaccine requirement for grocery store employees. Please see interview questions in Table A in the supplemental file. This activity was reviewed by CDC, deemed not research, and was conducted consistent with applicable federal law and CDC policy.^1^ Interviewers used a standardized script to obtain informed consent, explaining the study purpose, emphasizing the voluntary nature of participation and each interview question, and describing the potential risks and benefits.

### Participant recruitment

Two organizations were contracted to lead recruitment and conduct in-depth interviews with grocery store workers in the United States. Participants for in-depth interviews were screened to meet the eligibility criteria: ages 18 years and older; worked in grocery stores prior to the pandemic, throughout the initial phase of the pandemic (March-May 2020) and at the time of the interview (May 2021 – June 2022); persons of Black, Hispanic or Latino, Asian, and White race or ethnicity; not employed in senior management positions; and interfaced with the public for at least 75% of their shifts. Researchers used maximum variation purposive sampling [28] among participants whose occupation was listed as grocery workers to achieve a diverse sample by race and ethnicity, age, sex, union membership status, and U.S. geographic locations. Study participants were from the following states: California, Florida, Georgia, Idaho, Illinois, Kentucky, Michigan, Minnesota, New Jersey, New York, North Carolina, South Carolina, Ohio, Texas, Washington and West Virginia.

The data for this study were collected over three different time periods. During the first data collection period of May 2021-August 2021, 30 in-depth interviews were conducted, and potential participants were identified in one of three ways: (1) via existing local contacts, (2) via snowball recruitment, and (3) via in-store direct recruitment of workers. The remaining 45 interviews were conducted during August 2021 through December 2021 and in June 2022. For these 45 interviews, a national recruitment firm recruited 39 eligible interview candidates by distributing an electronic screener for study eligibility to 24,491 individuals in their network. The authors partnered with a community-based organization serving Southeast Asian persons. This partnership allowed the distribution of the screener to workers with an existing relationship with the organization. The community-based organization successfully recruited 6 workers after approaching 14 individuals. Study participants worked in a variety of grocery/food retail settings, including wholesale warehouses, “super stores” selling groceries and non-food products, traditional grocery stores, and Asian markets. Initially, the plan was to conduct 80 interviews in total. However, due to persistent challenges in recruiting Southeast Asian workers and the team’s unanimous consensus that further interviews were no longer providing new insights, and that saturation had been reached, recruitment was concluded after 75 interviews.

#### Interview procedure

A total of six trained qualitative researchers with expertise in public health, including four of the co-authors of this paper, facilitated the online interviews via Zoom using a standard semi-structured interview guide that we collaboratively developed (Table A in the supplemental file). During the first two data collection periods, a pilot in-depth interview was conducted with one grocery worker before refining and fielding the guide for the remaining interviews. Interviews were conducted in English and Khmer (Cambodian). Interviews in English were professionally transcribed. For the interviews in Khmer with an interpreter present, the research team prepared detailed notes based on the audio recording where the translator provided responses in English during the interview itself. Interviews typically lasted 90 min each, and participants received a $60 incentive for their time.

### Analysis

The authors used Rapid Turn-Around (RTA) qualitative data analysis [29] to understand the knowledge, attitudes, and barriers influencing uptake of the COVID-19 vaccine among grocery store workers. RTA is ideally suited for research projects from which policy makers, funding agencies, and government officials must quickly obtain insights needed to make timely decisions regarding strategy and practice [29, 30]. RTA techniques are adaptable and appropriate when the time available for analysis is limited to weeks or months and formal qualitative coding (e.g., using NVivo) would be time and/or cost prohibitive [31, 32]. For the current study, research findings were needed urgently to inform new products and support the U.S. COVID-19 response. RTA techniques have been deployed rigorously and lead to high-quality, impactful published research [33–35].

The RTA process for this study entailed several steps. First, one of the authors developed a template to condense and categorize the interview data into specific domains that aligned with the areas of inquiry. The template also included sections for analysts to identify key interview themes that did not align well with the predefined study domains, compelling quotes, and observations regarding participants’ nonverbal communication. Five study analysts piloted use of the template for condensing and summarizing participants’ interview responses for the same interview transcript and then met to review their work. Based on differences observed across the analysts, the team made minor changes to the template and developed more explicit instructions regarding how the template should be used. The five analysts then repeated the exercise with a second interview transcript, and after comparing their work, agreed that they had reached alignment in their analytic approach. At this point, the analysts finalized the template.

Analysts divided responsibility for independently populating templates for the remaining interview transcripts. Although these remaining templates were not reviewed by other analysts, the team met throughout the analysis process to discuss challenges they faced in summarizing the data and align on solutions.

Once all interviews were summarized in the populated templates, a subset of the analysts copied content from each domain of the transcript into matrices in Microsoft Excel. Unlike the populated templates, which contained data from a single interview, the matrices allowed the analysts to review summarized data across the interview discussions and according to participant characteristics, which were merged into the matrices from recruitment data files. The matrices were finalized following a peer review.

All five analysts used a structured process to distill themes from the matrixed data. First, analysts divided the matrices by interview domain and independently reviewed associated interview content to identify initial themes. The analysis team then met over several weeks to share the themes they had identified and refine them based on group discussion, as fellow analysts had insights from their experience facilitating the interview discussions and reviewing the interview transcripts. Between meetings, analysts further refined their themes by going back to the original transcripts for confirmation or clarification. Throughout this period, analysts also evaluated which interviews aligned with the themes they identified and located illustrative quotes from the interview transcripts.

The final themes from this analysis are presented as applying to varying proportions of participants, including very few (less than 10%), few (10–19%), some (20–39%), about half (40–59%), many (60–74%), or most participants (75% or more). The authors regard these approximations as helpful for describing patterns within the study sample and demonstrating rigor. However, exact percentages are not shown in acknowledgement of longstanding debates in qualitative research regarding whether it is appropriate to quantify qualitative findings [36, 37]. For this particular study, the authors caution readers against drawing conclusions about grocery workers overall based on the frequency of themes, as this study was not designed for generalizability, unstructured responses were occasionally challenging to classify with confidence, interviewers did not phrase protocol questions the exact same way across interview sessions, and the frequency of themes does not necessarily imply anything about their importance.

## Results

Of the total 75 study participants, 39 (52%) were female and 36 (48%) were male (Table 1). Participants identified as Black (*n* = 19, 25%), Asian (*n* = 16, 21%), White (*n* = 22, 29%), Hispanic or Latino (*n* = 16, 21%) and multi-racial (*n* = 2, 3%) and were of the following age categories: 18–34 years old (*n* = 28, 37%), 35–49 years old (*n* = 20, 27%) and ≥ 50 years old (*n* = 27, 36%). Two (3%) participants spoke in Khmer for their interviews; all other participants spoke in English.

**Table 1:** Demographic characteristics of study participants by vaccination status

Characteristics	*N*	Vaccinated *N* (%)	Unvaccinated *N* (%)
Overall	75	59 (79%)	16 (21%)
Sex			
Women	39	29 (74%)	10 (26%)
Men	36	30 (83%)	6 (17%)
Race/Ethnicity			
Asian	16	14 (88%)	2 (13%)
Black or African American	19	14 (74%)	5 (26%)
Hispanic or Latino	16	13 (81%)	3 (19%)
White	22	16 (73%)	6 (27%)
Multiracial	2	2 (100%)	0 (0%)
Age Group			
18–34 years old	28	22 (79%)	6 (21%)
35–49 years old	20	17 (85%)	3 (15%)
50 + years old	27	20 (74%)	7 (26%)
Insurance Status			
Employer	38	28 (74%)	10 (26%)
Private	19	16 (84%)	3 (16%)
Medicare/Medicaid VA	7	7 (100%)	0 (0%)
None	11	8 (73%)	3 (27%)

### Vaccination behavior and demographics

Table 1 shows the number and percent of participants by vaccination status and personal characteristics. Among the 75 grocery workers interviewed,79% (*n* = 59) were vaccinated and 21% (*n* = 16) were unvaccinated. Within the vaccinated group, 2 grocery workers had received their first dose of the vaccine and were scheduled to receive their second dose sometime after the interview. In the unvaccinated group, 2 workers were scheduled to receive the COVID-19 vaccine at a later date. Among Asian grocery workers, 13% (*n* = 2) were unvaccinated; among Black grocery workers, 26% (*n* = 5); among Hispanic or Latino grocery workers, 19% (*n* = 3); among White grocery workers 27% (*n* = 6); and neither of the two multiracial workers were unvaccinated (*n* = 0, 0%). By age, 26% (*n* = 7) of older (≥ 50 years) workers were unvaccinated; 15% (*n* = 3) of middle-aged (35–49 years) workers; and 21% (*n* = 6) of younger workers aged ≤ 34 years. 26% (*n* = 10) of women were unvaccinated, and roughly 1 in 6 of men interviewed were unvaccinated (*n* = 6, 17%). Among workers who had insurance through their employer, 26% (*n* = 10) were unvaccinated; among workers who had private insurance, 16% (*n* = 3); among workers with Medicare/Medicaid/VA, 0% (*n* = 0); and among workers with no insurance, 27% (*n* = 3).

The following two sections (Sect. Factors influencing vaccinated workers decision to get vaccinated and Factors influencing unvaccinated workers’ decision to not get vaccinated) identify the most common reasons why workers said they did and did not get the COVID-19 vaccine (Table 2). These findings are presented in order of frequency mentioned. Workers often provided multiple explanations for their decisions and different themes could thus apply to any one participant.

**Table 2:** Factors considered when deciding whether or not to get vaccinated by grocery store workers against COVID-19

Reasons* for getting vaccinated, among vaccinated workers (*n* = 59)	Reasons* for not getting vaccinated, among unvaccinated workers (*n* = 16)
1. Protection of family members, especially those that are at greater risk for COVID-19 (*n* = 28, 47%)	1. Possible side effects (*n* = 12, 75%)
2. Protection of selves from exposure to COVID (*n* = 25, 42%)	2. Belief that vaccine production was rushed, and ingredients were unknown (*n* = 9, 56%)
3. Trust in the science behind the vaccine/believing the vaccine is safe (*n* = 19, 32%)	3. Belief that immune system was already strong enough, sometimes as a result of prior COVID infection (*n* = 4, 25%)
4. Belief that COVID-19 as greater risk than vaccine side effects (*n* = 17, 29%)	4. Belief that vaccine is ineffective because you can still catch COVID and also would need a booster (*n* = 3, 19%)
5. Waited and saw others (e.g., public, family, friends, co-workers) who got vaccinated were ok (*n* = 9, 15%)	5. Negative comparison to flu vaccine (e.g., flu shot did not work; bad side effects from flu shot; will need booster like flu shot) (*n* = 3, 19%)

*Self-reported reasons

### Factors influencing vaccinated workers decision to get vaccinated

#### Protecting family members

Among 59 vaccinated grocery workers, the most common factor taken into account when deciding whether or not to be vaccinated was the desire to protect family members, particularly those who were at increased risk for severe COVID-19 including: children, elderly family members, and family members who were immunocompromised due to disease or an organ transplant. This included both family living in their home as well as extended family members whom they hoped to see at social gatherings. One participant shared that she got vaccinated because “*I wanted to keep myself and my family safe…My dad’s on chemo forever. So*,* anything that we could do to keep him safe is #1.”* Another participant said that her family was already vaccinated, and she *“wanted to contribute to the overall health of everyone in the household.”*

#### Protecting selves from severe COVID-19

The desire to protect their own health was the second most commonly mentioned consideration for vaccinated workers. One grocery worker explained, *“I just got it [the vaccine] because people are dying. If I don’t want to end up the same way too*,* then I might as well just get [the vaccine].”* A few vaccinated workers shared that they had a terrible experience being infected with SARS-CoV-2 prior to the vaccine being available and they wanted to be vaccinated to protect themselves from such severe illness in the future. One participant stated, *“I remember the way I was feeling when I was sick*,* and it was horrible*…*I never want to be that sick again*. *So that was kind of the pusher that made me certain I wanted to get vaccinated.”* A few others were high-risk for severe complications from the virus (e.g., immunocompromised, diabetic, cancer survivor) and viewed the vaccine as adding a much-needed layer of protection for their immune systems. The protection from the vaccine also translated to increased freedom for a few participants, who described being able to travel, attend concerts, or socialize with decreased concern about getting very sick.

#### Trusting the science behind the vaccine

Some vaccinated workers stated that they “trusted the science” behind the vaccine and believed that the vaccine was safe. One participant summarized, *“The majority of science was on the side that it [COVID-19 vaccine] was safe. I saw how it was made and saw it’s perfectly fine*,* no real concerns there.”* Another said that even though COVID-19 vaccine production seemed rushed, *“I know they’ve gone through the steps to make sure it’s a good thing.”*

#### Believing the vaccine is safe

Vaccinated workers also took into consideration the potential side effects when considering whether to get vaccinated; they decided the protective benefits outweighed the risks (“*I’d rather risk the vaccine than risk the virus*”), especially with the uncertainty of having a mild case or ending up with long COVID. One participant said that having mild side effects from the vaccine, such as chills, was “a small price to pay.” In a couple of cases, participants felt they would not be personally impacted by what they believed to be side effects. For example, one grocery worker said that she heard the vaccines can cause blood clots, but because her blood “does not clot well” she was not worried this could happen to her. Another worker said that she heard the vaccine can cause infertility, but she “was done having kids” so that would not have stopped her from getting vaccinated.

#### First assessing others’ vaccine experiences

A few vaccinated workers talked about waiting and seeing how family members, friends, coworkers, or even the general public responded to the vaccine before accepting that it was safe and deciding to get the COVID-19 vaccine. One grocery worker said that she got the COVID-19 vaccine *“because a lot of my coworkers around me were getting it*,* and they didn’t grow a third arm or anything*,* so I was like*,* ‘Okay*,* well*,* at least immediately I shouldn’t see any terrible side effects… [so I] just went and did it in good faith.”* Another participant said he waited *“a few months*,* to make sure everything was good”* before getting vaccinated. One of the other participants said he felt “safe enough” getting vaccinated once he saw that over 100 million people had gotten the COVID vaccine.

### Factors influencing unvaccinated workers’ decision to not get vaccinated

#### Possible side effects

The most common factor considered by the 16 unvaccinated workers when evaluating whether to get vaccinated was the possible long-term and short-term side effects or outcomes associated with receipt of the vaccine. Concerns included blood clots and experiencing symptoms that mimic COVID-19, though most participants spoke of side effects in general terms. For example, one worker shared, *“To me*,* it’s an experimental drug… They haven’t done enough research to know how it’s going to affect me*,* not just short term*,* but also long term*.” A second worker who was immunocompromised perceived the possible side effects as too risky for her body. Another worker was suspicious that the vaccine may kill you, saying, *“Then you hear somebody get [got] the vaccine and they passed away. But was it from the vaccine or something else? You don’t know*.” Another participant was worried that if they were to experience severe side effects, they would have no course of legal action: *“You can’t hold anyone liable if something happens to you after getting the vaccine. Nobody’s liable.”* Similarly, a worker was concerned about one vaccine being recalled and had also read that a lawsuit was filed against another COVID vaccine manufacturer; she worried about her own safety and reaction to the vaccine. A few participants were worried that they would be forced to miss work due to adverse side effects from the vaccine. One explained, *“I do know people personally that have had … their COVID shot*,* and they’ve had an adverse reaction to it*,* to where it took them out of work for like a few weeks. And it’s like*,* I’ve already had COVID*,* it already took me out for a couple weeks. I don’t want to get this COVID shot*,* and then I’m out of work for a few weeks*,* and I’m not being covered by my job.”* Another unvaccinated worker stated, *“I don’t want to have to miss work because I take it [COVID-19 vaccine]”*; the vaccine was perceived as an unnecessary inconvenience.

Within the unvaccinated study participants, we identified sex differences in concerns regarding side effects. Most unvaccinated women expressed concerns about side effects, compared to about half of the unvaccinated men. Specific side effects of concern reported by women were related to reproduction, including fertility issues, heavy menstrual periods, and severe menstrual cramping. One female worker shared, *" I heard that*,* not only heard but read*,* that it has effects on the women’s reproductive system. It can potentially make you infertile. So that’s a big no-no for me because maybe someday I want more kids.”*

#### Rapid vaccine development

More than half of the participants who were unvaccinated were hesitant to get the vaccine because of concerns associated with the rapid development of the vaccine and its composition. One participant said that the vaccine was not yet [FDA] approved. Among those who expressed concern about the vaccines’ ingredients, most said that they did not know what was in the vaccine and one participant stated that fetal tissue was in the vaccine. One unvaccinated grocery worker summarized her perspective of the COVID-19 vaccine’s development process: *“They just threw something together and they’re saying*,* ‘Boom! This is it.‘”* Others said they did not want to be “guinea pigs” with the “experimental” COVID-19 vaccine. Another said, *“I feel like I’m putting something into my body that I’m not 100% sure of what I’m putting into my body.”* An unvaccinated worker explained, *“There is a lack of knowledge on the virus*,* which translates to an extreme lack of knowledge on the vaccine”*; of particular concern was that the vaccine had been developed in less than 12 months, compared to the standard multi-year cycle. One participant was skeptical because of the variety of vaccines available, including the single dose version and the more common two-dose versions.

#### Belief in natural immunity

Some of the unvaccinated workers felt that their immune system was strong enough to fight COVID-19 without a vaccine. One participant said, *“In general*,* I think it’s just like any other vaccine*,* or shot*,* that’s going to strengthen your immune system. It’s like the common cold or the flu. They’ll give you the shot to strengthen your immune system*,* but I think mine’s strong enough already….”* Most of these unvaccinated workers reported they were previously infected with SARS-CoV-2 and felt that their bodies already had antibodies to fight the virus. One participant summarized, *“Well*,* for us [her and her husband]*,* we figured we’ve had COVID*,* so I have the antibodies in me and I think it’s been said that if you had COVID you have more of the antibodies than the actual vaccine that gets shot up in your arm. If it’s already in me*,* why should I have to get it again?”* The general consensus was that their previous infection(s) provided them with the same antibodies they would get from a vaccine, rendering the vaccine pointless.

#### Belief that vaccine is ineffective

A few unvaccinated workers perceived the vaccines as ineffective. The primary rationale used for this sentiment was that you could still become ill with COVID-19 after being vaccinated and you would still need a booster after being vaccinated. Workers were skeptical about the vaccines’ effectiveness in part because of the frequently changing public health guidelines. One worker, who had previously had COVID-19, shared her thought process: “*At first*,* it was like if you got [the vaccine]*,* it protected you against [COVID-19]. Now*,* it’s like well no*,* it doesn’t. Well*,* now there’s a booster that needs to come out. Now*,* even if you had [the vaccine] and had the booster*,* you still have to wear the mask…The information keeps changing and that’s what’s steering me more away from it…I was leaning toward it*,* but then as the information evolved and said*,* ‘Well*,* there’s no guarantee even after you get the vaccine that it protects you more than - I don’t know what it was back then - four months.’ It’s like*,* okay*,* well*,* the people that originally got it when it came out [in] June*,* now we’re in October - it’s worn off. It’s no longer effective. The information keeps changing. To me*,* that just makes me even more leery.”*

#### Negative comparison to flu vaccine

A few workers who were unvaccinated found it off-putting that the COVID-19 vaccines were being compared to flu vaccines (e.g., by their pharmacist), and that factored into their decision not to be vaccinated. One grocery worker experienced bad side effects from the flu shot a decade ago and has since avoided getting vaccinated. Another said, *“I’ve never had the flu shot because I feel like that’s not for me. I don’t know why*,* but it’s something in me telling me I don’t need it. Maybe I do*,* but I’ve never had the flu shot. So*,* I’m working on this [COVID-19] vaccine [in the same way] to see if I need to take it.”* A few workers were frustrated that a COVID-19 booster is needed, like the annual flu shot, which made it seem ineffective (as reported above).

### Employer support

#### Benefits provided by employers

Overall, vaccinated and unvaccinated workers received similar benefits from their employers. Comparable proportions of vaccinated workers and unvaccinated workers received hourly pay raises (long term hourly wage increases). Most unvaccinated workers had paid sick leave (which sometimes included time spent in quarantine) as well as flexible scheduling options, and many vaccinated workers reported having these benefits. However, only a few unvaccinated workers received hazard pay (short term pay raises or bonuses) as opposed to about half of vaccinated workers. Workers generally seemed satisfied with the benefits provided. One stated, *“They did the best of their abilities given the situation that was unfolding on a daily basis.”* The opposing viewpoint was that a few staff worked while sick to avoid loss of income: *“They didn’t do anything to support the staff. They just tried to make it look like they were safe for the public…We had several people who were sick and lost their taste and smell and all that stuff*,* but they still worked because they couldn’t take off because they couldn’t afford [it]…So*,* they just worked through it”* Most unvaccinated workers had paid sick leave (which sometimes included time spent in quarantine) as well as flexible scheduling options, and many vaccinated workers reported having these benefits.

#### Incentives to be vaccinated

Some of the vaccinated and unvaccinated grocery store workers reported that their employers offered an incentive to be vaccinated. This included cash bonuses from $40 to $120; paid time off to get vaccinated and recover from any side effects; gift cards to the grocery store or a restaurant delivery service; and in two cases, a raffle for large prizes, including a new iPad or $1,000 cash. Workers generally spoke favorably of being provided an incentive, regardless of vaccination status. Only one participant - who was unvaccinated - described the provision of incentives in a negative way, saying it felt like the store was trying to bribe staff to be vaccinated: “*They offered like $120*,* and in all honesty that kind of freaked me out. I was like*,* ‘Oooh*,* if you’re paying me to take this*,* I don’t know.’…I understand what they’re trying to do – they’re trying to encourage receiving [the vaccine]. But…it felt like a bribe in a way*.”

### Attitudes about employer vaccine requirement

About half of the vaccinated workers supported an employer vaccine requirement and some were opposed to it. Those in support thought that “*the greater good is more important than personal freedom*” and such a requirement would *“help save lives”*, help things get *“back to normal*”; and protect elderly workers. Some vaccinated grocery store workers even felt that *all* essential workers should have a vaccine requirement. A couple of participants stated that an employer was entitled to implement a vaccine requirement, and that workers who had a problem with it “*could go get another job*.” Vaccinated workers also perceived that by implementing a vaccine requirement, employers would be showing that they cared about their workers’ well-being. A few vaccinated workers expressed conditional support of a storewide vaccine requirement, saying that it was a good idea as long as religious and medical exemptions were considered. Among those vaccinated workers who opposed the requirement, freedom to choose whether or not to be vaccinated was the most common reason for their opposition. One worker summarized, “*Well*,* no. I don’t think that you should make it mandatory for anybody to tell someone to do anything. No. That should be a choice. That’s something that we are granted as humans*.” Another participant compared requiring vaccination to forcing diabetics to use insulin. “*That’s like telling a person who has diabetes that they have to take insulin. I just don’t think it’s any of [the store’s] business*.”

All unvaccinated workers opposed a vaccine requirement. These workers emphasized the importance of personal choice and didn’t want “*to feel like [the vaccine] is something that’s being forced onto us.”* One unvaccinated worker worried that requiring the COVID-19 vaccine could lead to a slippery slope: “*I’d hate to be an employee there [at a store with a vaccine requirement]. I don’t agree. I think it should be optional. It’s our body*,* our choice. If we let them mandate this*,* what’s next?”* A few said they would be “disappointed” if such a requirement was put in place and thought that workers would quit because of it. Only one unvaccinated worker was receptive to eventually requiring the vaccine “*if it’s proven to help not to get COVID*” but thought that it was too early to make that determination. Among both vaccinated and unvaccinated workers, a few participants said their store was already requiring employees or new hires to be vaccinated.

### Sources of vaccine information

When asked about where participants received information regarding COVID-19 vaccines, grocery store workers identified various sources (Table 3). Vaccinated workers identified CDC and the news (print, tv, radio, or online) as the sources they most often turned to for information. Online sources of news included articles that appeared in newsfeeds and news outlet apps (e.g., BBC, CNN, Yahoo News, Fox News). Unvaccinated workers identified the news and medical professionals as the sources they most often used for information about the COVID-19 vaccines. Table 3 shows the most referenced sources of information by vaccinated and unvaccinated grocery store workers and includes illustrative quotes. Both vaccinated and unvaccinated participants often referenced similar sources for information. However, unvaccinated participants were less trusting of the information that was shared due to changing COVID-19 information and guidelines over time.

**Table 3:** Information Sources Consulted by Vaccinated and Unvaccinated Grocery Store Workers for COVID-19 Vaccines, Ranked by Preference

	Vaccinated Workers (n=59)		Unvaccinated Workers (n=16)	
Rank	Source of Information	Quote	Source of Information	Quote
1.	*CDC (*n*=33, 56%)	*“I go to the CDC website. Where you should go.”*	News (*n*=11, 69%)	*“But then there's so many news outlets, depending on what news channel you watch that contradicts what the CDC is saying… Even Fauci has gone back and forth, back and forth. It keeps changing. So it's like okay, you had us in the beginning, but now you have to change this so many times. It's like, I don't believe anything...”*
2.	News (*n*=29, 49%)	*“* *A lot of the information I got on it was from our local news.”*	Doctor or pharmacist (*n*=9, 56%)	*“* *They [store pharmacists] did try to press it on us to get it. They were like, ‘It's just like your annual flu shot. Get the vaccine.’ They told us about it, but I haven't done much research on my own...”*
3.	Friends or family (*n*=17, 29%)	*“Friends and family said the COVID shot had benefits, it was positive. They shared information.”*	CDC (*n*=8, 50%)	Has gone to the CDC website and *“it’s ever-changing.”*
4.	Doctor or pharmacist** (*n*=16, 27%)	*“* *I contacted my physician, my mom contacted her [lung] transplant team over at [the health clinic] and they all were like, ‘Yes, you need to get the vaccine. You especially need it. It will protect you. It's a must.’ And they have it readily provided for her, ready for her because of the fact that she's a transplant patient. So that's all I really need to know. When I have these people who have already saved my mom's life and they're saying like, ‘Hey, you should do this as well.’ Let's do it.”*	Friends or family^¥^(*n*=4, 25%)	*“* *I'm a little worried. I already have a son. I just plan on having another and I've heard from many friends and another employee - she's planning to get pregnant as well - and her OB actually asked her not to get vaccinated for her and her husband. So, we're in that same boat.”*
5.	Internet searches** (*n*=16, 27%)	*“[I look at] whatever’s online that’s provided…Just look up COVID-19. Google ‘COVID-19.’ There it is right there.”*	Internet searches ^¥^(*n*=4, 25%)	*“I usually go online, and I will Google the vaccines and I try to read various reports and get the information I can online from what would be, or what I would consider to be medical websites. Not opinions or, ‘my cousin, Joe.’"*
6.	Local or state health department (*n*=15, 25%)	Participant and co-workers used their state’s Department of Health website:* “It was the .gov [website] to look at where the vaccines were available, which sites were doing it. And they were very, very detailed as to the different [brands of vaccines]...The Johnson & Johnson, the Moderna, the Pfizer.”*	Social media^¥^(*n*=4, 25%)	*“S* *ometimes [information about the vaccines] pop up in my news feed in social media [Facebook] and sometimes in YouTube.”*

*Centers for Disease Control and Prevention

** Among vaccinated workers, doctor or pharmacist consultations and internet searches were equally popular sources of information

^*¥*^ *Among unvaccinated workers, friends or family, internet searches and social media were equally popular sources of information*

### Preferred mode of health communication

About half of the vaccinated and unvaccinated workers preferred in-person work-related communication (e.g., meetings, one on one talks, huddles, manager speaking) as well as electronic work-related communication (e.g., email, video, text, website) of health information related to COVID-19. However, in-person communication was more popular among vaccinated workers whereas electronic communication was more popular among unvaccinated workers. Among both vaccinated and unvaccinated workers, in-person communication or electronic communication was more popular than written communication (e.g., pamphlets, flyers, posted notices, materials). More than half of the participants shared that they would have liked to receive additional information from their employers about various topics including COVID-19 vaccination. Among participants who said they had received information from their employers, about half said information was provided by printed documents and handouts, fact sheets, and signage, but most workers preferred to receive information in person either one-on-one or in small group meetings, or electronically through text messages or emails. A few workers thought meetings were a better way to receive information because the manager leading the meeting lends authority to the topic area, but also because some people do not like to read, or they may ignore pamphlets or emails.

Most workers, irrespective of racial and ethnic category, preferred receiving information during in-person meetings. For instance, most Asian workers received information verbally during meetings or in conversations (e.g., from a manager) and one Southeast Asian worker from a small market noted that the advantage of meeting in person was to hear the information in their native language since some workers do not speak English. Another worker who is Asian said that staff did not use technology, so in-person information was the best way to communicate. Some workers indicated that in-person meetings facilitate a sense of community, allow them to ask questions, learn from others’ experiences and is a sign of care and concern for the staff. Written communication was the least preferred mode of communication irrespective of racial or ethnic background.

Table 4 presents the communication modes in order of popularity, by age group. For all age groups, information shared virtually (e.g., via store website, video) was the least preferred mode of communication. While the top choice of workers who were middle-aged (35–49 years old) and older (50 years and older) was to receive COVID-19 health communication in-person and during staff meetings, the younger workers’ (18–34 years old) top choice was to receive information via email or text. Several younger workers noted that “people are on their phone anyway” so it made sense to them to send information via text or email.

**Table 4:** Preferred employer communication modes for COVID-19 vaccine information by age Group, ranked from most to least common

Rank	18–34 years old (*n* = 28)	35–49 years old (*n* = 20)	50 years and older (*n* = 27)
1.	Emails/texts (*n* = 14, 50%)	In-person and meetings (*n* = 10, 50%)	In-person and meetings (*n* = 21, 78%)
2.	In-person and meetings (*n* = 13, 46%)	Emails/texts (*n* = 8, 40%)	Printed documents, signage, handouts, fact sheets (*n* = 13, 48%)
3.	Printed documents, signage, handouts, fact sheets (*n* = 9, 32%)	Printed documents, signage, handouts, fact sheets (*n* = 8, 40%)	Emails/texts (*n* = 6, 22%)
4.	Virtually online, video, store website (*n* = 6, 21%)	Virtually online, video, store website (*n* = 5, 25%)	Virtually online, video, store website (*n* = 6, 22%)

## Discussion

COVID-19 vaccination is the most effective method for reducing the risk of getting severe COVID-19, including decreasing risk of hospitalization or death from COVID-19 [38, 39]. However, social, economic, cultural, and political factors influence how individuals perceive the vaccines, and their decision to vaccinate or not [40]. In this study, motivations to vaccinate centered on protecting oneself and family members, while vaccine hesitancy was driven by concern about side-effects, skepticism about the vaccine’s rapid development, and exposure to misinformation and workplace barriers. Using the World Health Organization (WHO)’s “3 Cs” model of vaccine hesitancy (Confidence, Complacency and Convenience) [41] provides a framework for understanding how individual, structural and informational factors interact to shape workers’ attitude and behaviors towards vaccines.

### Confidence: trust in vaccine safety, effectiveness and information source

Concerns about vaccine safety and potential side-effects played a central role in vaccine hesitancy. Many unvaccinated workers were concerned about the potential short- and long-term side effects, with women in particular, concerned about potential impact on fertility. All but one of the unvaccinated women in the study reported these as a major barrier to vaccine uptake. Interestingly, although the American College of Obstetricians and Gynecologists (ACOG) recommended pregnant women and those considering pregnancy to get vaccinated [42], this fear of side effects persisted among the female participants who were planning to get pregnant. The side effect of concern most frequently mentioned was the belief that vaccines can potentially cause infertility. This was consistent with a previous survey of unvaccinated U.S. adults conducted in 2021 that reported 38% of unvaccinated participants believed that COVID-19 vaccine could negatively impact an individual’s fertility [43, 44]. Another study found 500% increases in infertility-related Google searches after the emergency use authorization (EUA) of Pfizer-BioNTech coronavirus vaccine [45]. Peer-reviewed research so far shows no evidence that COVID-19 vaccine causes infertility among vaccinated women or men [46]. Another prospective cohort study of 2,126 females aged 21–45 years found no adverse association between COVID-19 vaccination and fertility [47]. Some of these fertility-related concerns may have been due to misinformation, such as a false report that stated that getting the COVID-19 vaccine would cause an immune response against a protein found in the placenta and jeopardize fertility [48].

Skepticism about vaccine effectiveness also lowered the confidence. Some participants believed that vaccine development was rushed, leading them to question its effectiveness and safety. The skepticism about vaccine effectiveness among some study participants was also partly due to the changing public health guidelines related to the vaccine, limited knowledge about vaccine ingredients, and the need for a booster shot after the initial vaccination. Some study participants also linked the COVID-19 vaccine to the influenza vaccine (flu shot) that they believed to also be ineffective, has bad side-effects, and is needed annually. Some workers who received the COVID-19 vaccine waited to see its effects on others before getting vaccinated themselves, indicating concerns about vaccine safety. Our findings were consistent with previous studies conducted in U.S. general population [49, 50].

Misinformation also contributed significantly to declining confidence. Despite the CDC website being a common reported source of information among both vaccinated and unvaccinated workers, misinformation was still prevalent, highlighting the need for accurate, unbiased, and balanced reporting of research findings by journalists and public health entities. The prevalence of conflicting information on news channels and social media platforms made the population vulnerable to misinformation, which might be addressed by the increased promotion of evidence-based research [51, 52]. Furthermore, social media has made it easier for rumors, misinformation, and disinformation to spread quickly [53, 54]. A study by Yang et al. (2021) found that 46.6% of the Facebook posts that discussed the COVID-19 vaccine were misinformation, and 47.4% were fact-checking posts defined as any public account including both individual and organizational accounts that posts factual information about COVID-19 vaccine or posts debunking information about COVID-19 vaccine information [54]. These findings emphasize the need for evidence-based research, a deeper understanding of how digital social networks operate, and strategies to fact-check information and address rumor mongering, particularly given the rise of multiple social media platforms.

### Complacency: perceived risk and belief about immunity

Some unvaccinated workers’ decision was influenced by belief that immunity from a prior SARS-CoV-2 infection or innate immune response was sufficient to prevent COVID-19 from developing and that vaccines provided no additional benefit. However, recent research indicates that vaccine-induced immunity is more effective than immunity acquired through previous infection in protecting against laboratory-confirmed COVID-19 [55]. In addition, hybrid immunity, or immune protection received from one or more doses of the COVID-19 vaccine and having at least one SARS-CoV-2 infection, may provide additional risk reduction against the reinfection and hospitalization [56].

Some participants linked the COVID-19 vaccine to the influenza vaccine, which they believed to be ineffective, associated with bad side effects, and are required annually. Changing public health guidelines, limited knowledge about vaccine ingredients, and the need for booster dose after initial vaccination further contributed to skepticism about vaccine effectiveness.

### Convenience: workplace barriers and access challenges

Several workplace-related factors affected vaccination decisions. The absence of factors such as paid time off or sick leave or availability of flexible scheduling led workers to report concerns about losing income when getting vaccinated and managing potential side effects. These barriers may have been exacerbated by a lack of culturally and linguistically appropriate health communication. In combination, the lack of workplace benefits and tailored health communication may have forced workers to continue to work when they had COVID-19 related symptoms, caused delay in getting tested for SARS-CoV-2 and uptake of the vaccine. Our study findings were consistent with emerging literature on COVID-19 vaccine hesitancy among the general population [23, 38, 39]. Conversely, the knowledge that they could receive time off from work, whether paid or unpaid, in case they fell ill, could have somewhat reduced their motivation to get vaccinated. One vaccinated worker stated that paid sick leave for COVID-19 was only offered to vaccinated employees, a policy implemented by their store in an attempt to promote vaccine adoption.

Studies have shown that implementing paid sick leave policies can increase both influenza and COVID-19 vaccine uptake, thereby reducing population-level differences in vaccination coverage among low social economic status neighborhoods [57, 58]. However, as of 2022, nearly 25% of U.S. workers and more than half of the nation’s largest retail and food services companies’ employees did not have access to paid time off for sickness, vaccination, or medical care [59, 60]. A few unvaccinated workers in the current study were fearful of the potential side effects of the vaccine and having to take unpaid sick leave to deal with them.

The lack of culturally and linguistically appropriate health communication further shaped workers’ decisions. Unvaccinated grocery store workers in this study reported receiving limited or poor information about vaccine development, safety, and effectiveness. These communication gaps may have reduced understanding of vaccine information and contributed to delays in vaccine uptake.

Participants’ vaccination status did not show any discernible link with attitudes toward employer vaccine incentives. However, variations were observed in their attitudes towards storewide requirements and the sources they used to obtain information about the vaccine.

### Implications

Although vaccine hesitancy is not a new phenomenon, it has been exacerbated by social media platforms and ideological beliefs [61, 62]. In the digital age, individuals with similar beliefs can efficiently connect and self-organize across geographic regions, influencing public confidence and cooperation [62]. Grocery store workers often rely on digital media for health information and work in a fast-paced, public-facing environment and may be more prone to misinformation. Evidence-based social media interventions that specifically target and counter anti-vaccination content and combat misinformation are especially needed to build upon the public health gains achieved during the pandemic [61]. It is beneficial for these gains to be extended to all segments of the workforce.

Systematic monitoring of public attitudes and perception regarding vaccination on social media platforms could provide an efficient, unobtrusive and rapid surveillance mechanism enabling public health officials to identify emerging concerns among grocery store workers and other workforces and develop timely and evidence-based intervention [63, 64]. Real time “social listening data” [65] would complement the existing evidence and help improve the design of new, robust and appropriately targeted interventions [64]. 

Findings from this study underscore the importance of cultural and linguistic diversity in health communication efforts and ensuring consistent messaging for grocery store workers. Employer and public health communication efforts that lack cultural relevance or plain-language messaging risks excluding minority groups from having fair and equitable access to lifesaving and life-enhancing health information. Our findings demonstrated the likely benefit of culturally sensitive, multilingual, and age-specific health education tailored to appropriate literacy levels and delivered through preferred modes of communication for grocery store workers through trusted community members, such as community leaders and healthcare providers.

Future research may examine the role of workplace policies and employer-led interventions in shaping vaccine uptake among grocery store workers and other workforces, particularly how variation in access to paid sick leave or paid time off for vaccination or medical care influences workers’ decision. Prior studies have shown that paid sick leave can increase influenza and COVID-19 vaccination rates and reduce population-level differences in coverage [57, 58], yet many workers lack access to such benefits [60]. The importance of universal and equitable access to these workplace benefits merits further investigation. Additionally, future studies could examine how cultural and linguistic relevance of health communication influence workers’ understanding of vaccines and their decision to be vaccinated and how employer might tailor communication strategies to better meet their workers’ needs.

### Study limitations

This study has several limitations. First, data were collected at three different time points during the pandemic; shortly after vaccines became available (May 2021–August 2021), during the Delta variant wave and before boosters were available (August 2021–December 2021), and after the Delta and Omicron variant waves (June 2022). Limited time and resources did not allow the authors to stratify the sample to systematically contrast participants depending on interview timing. However, a study by Mayer et al., who examined vaccine hesitancy among grocery store workers using Arizona Frontline Workers Survey did identify variability in select themes based on when interviews were conducted [66]. Between July 2020 and January 2021, 17% (*n* = 125) of workers reported an increased level of hesitancy while 7% (*n* = 50) reported a decreased level of hesitancy and thus greater intent to get vaccinated [66]. Regardless of whether and how the themes identified here may have varied in relation to interview timing, we feel that all of the facilitators and barriers to vaccination described can be helpful for informing efforts to reduce vaccine hesitancy.

Second, the recruitment of workers who were East Asian was challenging. This demographic is quite small—grocery store workers are a subset of all retail workers, Asian workers are one of the smallest population groups within that subset of workers, and East Asian is an even smaller group—and many public health organizations and researchers have not developed the networks and language skills to effectively reach these workers [18]. Additionally, employing maximum variation purposive sampling introduces complexities in recruiting participants from certain ‘hard to reach’ populations due to the stringent recruitment criteria. However, the inclusion of East Asian workers was crucial for the research team due to significant levels of violence and discrimination experienced by this community during the COVID-19 pandemic [67, 68]. The data collection team worked to overcome this recruitment barrier by refining our inclusion criteria to include grocery workers who are Southeast Asian and by partnering with a small community organization that serves this population. The effort undertaken in including populations that are often dismissed as ‘hard to reach’ provided invaluable information and reinforces the importance of inclusion of diverse populations of workers in occupational safety and health research [18].

Thirdly, participants and interviews characteristics weren’t matched so there could be interviewer effects. The interviewer-interviewee discordance can increase social desirability bias where participants may be less willing to discuss sensitive topics relating to their personal characteristics with the interviewers in this study or may tailor their responses to conform to perceived social norms or interviewer expectations.

Finally, due to the qualitative nature of the study, the findings are not statistically generalizable. Voluntary participation may have introduced selection bias, which could limit the extent to which result reflects the attitudes, beliefs, behaviors, and preferences of grocery store workers more broadly. To address this limitation, we utilized maximum purposive sampling to intentionally recruit participants with diverse sociodemographic characteristics and workplace experiences, which helped ensure inclusion of broad range of perspectives. While this approach reduces, but cannot eliminate, selection bias, it enhances the credibility and interpretability of the findings by reducing the likelihood that the results reflect a narrow or homogeneous group.

The qualitative methodology employed for this study allowed us to probe deeper on workers’ perceptions related to COVID-19 vaccines than the alternative quantitative approaches. Qualitative approaches aim to capture the needs or concerns of the community in their own voice and from their own perspective. This is particularly important when interviewing individuals from communities that have been historically underrepresented in public health research because it helps to avoid the potential cultural bias within existing quantitative data collection instruments. Furthermore, it can help researchers and public health officials identify their unexamined cultural assumptions that may bias their understanding of the issues [69]. These data can inform the development of targeted interventions and materials that are culturally and geographically tailored to the needs of these workers. Qualitative research employing purposive sampling was particularly important during the pandemic due to the novel nature of the virus and vaccines and the limited capacity of public health organizations to work effectively with the diversity in the critical infrastructure workforce.

## Conclusion

Our findings support the critical need to reinforce the safety and effectiveness of COVID-19 vaccines while addressing the misinformation and disinformation that pervades a racially, ethnically, culturally, and linguistically diverse workforce responsible for maintaining the U.S. infrastructure. Although this study focuses on the COVID-19 pandemic, the findings remain relevant, as participants frequently compared COVID-19 vaccine to influenza vaccines highlighting their concerns for vaccine effectiveness and need for booster or repeated dosing. These comparisons suggest that vaccine attitudes may extend across immunization context, with implication for other vaccination efforts as well as response to future outbreaks. This study highlights the importance of addressing structural challenges faced by the grocery store workers including inconsistent access to paid sick leave, hazard pay and paid time off. These workplace conditions shaped workers’ decisions and in some cases limited their ability to seek testing and recover from illness without financial hardships. Lessons learned from these experiences underscore the importance of better understanding and addressing the needs of grocery store workers and other frontline workers, and of implementing supportive workplace policies that are applied uniformly across sectors rather than selectively, which may help reduce inequities in protection for workers during future public health emergencies. Our findings align with the World Health Organization’s framework for immunization campaigns that focus on ‘confidence, complacency, and convenience’: ‘Lack of confidence’ in the safety and effectiveness of the vaccine among the public and policymakers, ‘Complacency’ due to the perceived low risk of vaccine-preventable diseases (e.g., the belief that those with natural immunity don’t need the vaccine), and ‘Inconvenience’ in accessing vaccines (e.g., a lack of paid time off to get vaccinated) [41]. The workplace, with the support of employers, offers an invaluable opportunity to address factors related to vaccine uptake, ultimately promoting higher vaccine adoption.

## Supplementary Information

Supplementary Material 1.

## Data Availability

The datasets presented in this article are not readily available as data use is restricted to the project team to maintain participants’ confidentiality. Requests for access to the datasets should be directed to Dr. Harpriya Kaur, [wdo6@cdc.gov] (mailto: wdo6@cdc.gov) .

## Footnotes

1 See e.g., 45 C.F.R. part 46.102(l)(2), 21 C.F.R. part 56; 42 U.S.C. § 241(d); 5 U.S.C. § 552a; 44 U.S.C. § 3501 et seq.
