# Supplementary material for: Grocery store workers’ knowledge, attitudes, and barriers influencing uptake of COVID-19 vaccine in the United States: a qualitative study
Source: BMC Public Health. 2026 Apr 2;26:1546. doi: 10.1186/s12889-026-26684-y (PMC13170031; doi:10.1186/s12889-026-26684-y)
Supplement: Supplementary file 1 — Supplementary Material 1. [file 12889_2026_26684_MOESM1_ESM.docx]

**Supplement**

**Table A. Select In-Depth Interview Questions* with Rapid Turnaround Analysis (RTA) Domains.**

| **Select In-Depth Interview Questions*** | **RTA Domains** |
| --- | --- |
| Work experience during COVID | |
| 1. What, if any, information has your store/employer provided on COVID-19?   Was there any information specific to the COVID-19 vaccines?   - - 1. Probe for: safety, efficacy, accessibility   Was there any information specific to the Delta variant?   - - 1. Probe for: transmissibility, severity, testing, prevention   Was there any information specific to the COVID-19 booster shot?  In what format was the information shared (e.g., in-person, e-mail, paper handout, virtual meeting or forum)  How easy was this information to understand?  How easy was this information to incorporate into your daily activities? | Store/employer info on COVID |
| Delta variant | |
| Any probes explicitly asking about Delta (e.g., probes about whether/how things have changed due to Delta, like information shared, stress level, or COVID protocols/mask mandates) | Delta variant |
| Safety and prevention perception | |
| 1. a. Tell me more about what your store/employer does to protect you from getting infected at work? For example:   What safety measures has your store/employer implemented to protect workers?  *[Probe for: provided mask/face covering, gloves, face shield, limited shoppers in the store, temperature check, symptoms screening, required social distancing, installed clear plastic partition between customers and employees, required masks for both customers and employees, sanitized the store after closing]*  How and by whom were these measures enforced?  What measures did your store/employer put in place to prevent sick workers from coming to work?  *{Probe for: paid sick leave, administrative policies like scheduling changes, any incentives*]  Did your store/employer provide information on where you can get tested for COVID-19?  Did your store/employer provide information on where you could get vaccinated against COVID-19?   1. [If vaccines were available onsite] Did your store/employer help with scheduling appointments for staff to get vaccinated against COVID-19?   What else did your store/employer do to protect workers during the pandemic?  What changes have taken place with safety measures in your store since the pandemic started (March 2020) until now (with Delta variant)?  **Include if they do not have certain protective measures in place (e.g., no masking of customers, no staff temperature checks)** | Protective measures by store/employer |
| 1. How do you feel about CDC’s guidance on mask mandates? Specifically, the change in guidance over time.   For example, initially the guidance was that masks were not necessary, then masks were required, and then if you are vaccinated you don’t need to wear a mask, and now if you are fully vaccinated you should wear a mask indoors in public if you are in an area of high transmission, and if you are unvaccinated-continue to wear a mask. | CDC mask mandate |
| 1. What has your store/employer done to support you during this time (e.g., hazard pay/salary raise, provide access to free COVID-testing)? This can include just the beginning of the pandemic…   Have you been able to take paid leave if needed?   - - 1. Did you need to take paid sick leave during lockdown period (March – May 2020)?   Has your supervisor been flexible with your schedule to allow you to care for children or other family members if needed?  This could include changing the time of your shift or days of work, or even allowing you to leave during your lunch break to check on loved ones. It could also include taking leave from work to help care for one of your loved ones who became sick. *[Note to interviewer: this could also include being allowed to take leave under FMLA]*  Has your supervisor provided you with cleaning supplies to keep your work area clean, and the time to do so?  Has your supervisor provided you with any resources to help you cope with stress during this time (such as pamphlets on coping, access to an EAP employee assistance program, mental health benefits, or other?)  Has your supervisor been someone you can talk to about your cares and concerns as a worker?  Is your supervisor following the same practices as the store management? If not, how is it different?  **Include support not provided (e.g., no hazard pay, no paid time off)** | Store/employer support provided |
| 1. What COVID-related training has your store/employer provided?   What types of training have you received on how to protect yourself from getting infected (if required)? What type of training did you receive about the safe use of masks/gloves/face shields (if not already discussed)?  In your experience, do you feel comfortable with the training and information on COVID-19 safety that you received at work to keep yourself safe? If yes, how so? If not, what were you not comfortable with?  How was the training provided? (Online/ In-person)  How did you feel about the training? | COVID training provided |
| Personal experience with SARS-CoV-2 infection and death and seeking care | |
| 1. Have you or someone in your life – like a co-worker, family member, or friend – tested positive for coronavirus?   If yes, who?   - - 1. Did [you/they] test positive after being vaccinated against COVID?        1. If Yes, how did that make you feel about vaccination?     2. {if the interviewee or co-worker got infected then ask] How did your store/employer react?   How did it affect your work?  How did it affect your life, in general?   1. Has someone in your life – like a co-worker, family member, or friend – died from coronavirus?   If yes, who?  If you shared this with your store/employer, how did your store/employer react?  How did it affect your work?  How did it affect your life, in general? | Personal experience with SARS-CoV-2 infection and death |
| 1. If you had to seek medical care during the pandemic (like going to a doctor), how do you pay for it?   Private insurance –   - - 1. Probe for- store/employer based, direct purchase health insurance, own employment-based health insurance.     2. Does your store/employer support the costs of your insurance?   Federal/state government program – e.g., Medicaid, Medicare, Tricare, VA Health care, State Health Insurance Assistance Plan (SHIP)  Out of pocket | Payment for medical care |
| COVID-19 vaccine-related knowledge, attitudes, and behaviors | |
| 1. Did you receive the COVID-19 vaccine yet?   Why [or why not]?  [If Yes] Do you plan to get the booster shot when it becomes available to you?  - Why or why not?  [If No] Do you plan to get vaccinated?  **[All] What factors did you weigh when considering whether or not to get vaccinated against COVID-19?** | COVID vaccination status and feelings |
| 1. Has your store/employer asked about your vaccination status?   If so, were you asked to show your vaccination card as proof?  Has your store/employer provided any incentive (e.g., gift card, bonus check) for staff who have gotten vaccinated against COVID-19?   1. How do you feel about COVID-19 vaccines?   Do you have any concerns related to COVID-19 vaccine? (e.g., side effects of the vaccine, safety, effectiveness)  Do you prefer one brand of vaccine over the others (Pfizer, Moderna, or J&J)? Why?   1. How do your co-workers feel about the vaccine? 2. What are some reasons you are hearing why your coworkers want or DO NOT want to get vaccinated? |  |
| 1. Are staff at your store required to be vaccinated against COVID-19? 2. How do you feel about stores requiring their staff to get vaccinated? 3. What are your thoughts on whether the COVID-19 vaccine should be mandatory?   For other essential workers  For the general public | COVID vaccine requirements/ mandates |
| 24ii. Where do you get your information about vaccines? Please be specific (e.g., specific news station, specific social media platform).   1. What did you hear about the safety of the COVID-19 vaccines?   Where do you go when you need more information about the COVID-19 vaccines?  Have you been to the CDC website? Or for your state or local public health department? | Sources of information about COVID vaccines |
| Information needs and recommendations related to COVID | |
| 1. What topics did you want to receive training on, that were not offered? 2. What information or training that has not been provided by your store/employer, but you think would help you the most to keep yourself safe during the pandemic? Probe for:    1. What topics would be most important to cover? e.g., vaccine information 3. What is the best way to share information about COVID-19? What way would work best for you and your co-workers? Probe for:   Brochure/Videos/Factsheets  Email/Text/Social media group post/Whatsapp  Pre-shift team meetings/All Hands store meetings/Town halls | Information and training needs and preferences |

**Only questions pertinent to the scope of this study are included here*
